# Telerehabilitation Following Stroke: Development of Training Content and Evaluation of an App-Based Training Program

**DOI:** 10.2196/77090

**Published:** 2026-03-31

**Authors:** Carina Ziller, Szabina Gäumann, Silya Lüscher, Nele Paulissen, Frank Behrendt, Zorica Suica, Björn Crüts, Luana Gamerschlag, Katrin Parmar, Hans Ulrich Gerth, Leo H Bonati, Corina Schuster-Amft

**Affiliations:** 1 Research Department, Reha Rheinfelden, Salinenstrasse 98, Rheinfelden, 4310, Switzerland, 41 618365382; 2 Physiotherapy Department, Reha Rheinfelden, Rheinfelden, Switzerland; 3 School of Engineering and Computer Science, Bern University of Applied Sciences, Biel, Switzerland; 4 Blended Clinic AI GmbH, Nuremberg, Germany; 5 Translational Imaging in Neurology (ThINk) Basel, Departments of Head, Spine and Neuromedicine and Biomedical Engineering, University Hospital, University of Basel, Basel, Switzerland; 6 Department of Medicine, University Hospital Münster, Münster, Germany; 7 Stroke Center and Department of Neurology, University Hospital Basel, Basel, Switzerland; 8 Department of Clinical Research, University of Basel, Basel, Switzerland; 9 Department for Sport, Exercise and Health, University of Basel, Basel, Switzerland

**Keywords:** telerehabilitation, stroke, app-based training, usability, blended care

## Abstract

**Background:**

Enhancing rehabilitation methods for patients with stroke is essential, particularly during the transition from inpatient to outpatient care. Digital applications are being developed to provide telerehabilitation programs. The existing virtual blended care platform Blended Clinic (Blended Clinic AI GmbH) offers app-based training for patients after a stroke and comprises 3 main components, including training, coaching, and monitoring.

**Objective:**

This study assesses the usability and user experience of the novel Swiss Tele-Assisted Rehabilitation and Training (START) program within the Blended Clinic platform in patients after stroke and therapists.

**Methods:**

The START program was developed within 3 workshops and an online survey. It contains 10 6-week exercise programs tailored to the levels of the modified Rankin Scale (mRS), 5 infographics, and 5 podcasts. Eight patients after stroke and 10 therapists took part in a single-center usability study. All participants were introduced to the Blended Clinic app and subsequently used it independently. The Blended Clinic platform, including the START program, was evaluated by both user groups based on the System Usability Scale (SUS) and the Mobile App Rating Scale (MARS-G). Additionally, feedback was collected, observations were documented, and program adherence metrics were calculated.

**Results:**

The mean SUS scores were 87.2 (SD 10.8) for 8 patients and 83.3 (SD 11.3) for 10 therapists. The MARS-G scores were 3.9 (SD 0.5) for patients and 4.1 (SD 0.4) for therapists for categories A-D. User experience was rated 4.1 (SD 0.5) for patients, while device usability was 3.8 (SD 0.8) for patients and 4.2 (SD 0.5) for therapists. Adherence to the training schedule varied among patients (16.7%-80% of the planned sessions) and was rather low for many of the patients.

**Conclusions:**

The START program, delivered via the Blended Clinic platform, was considered user-friendly and received good usability ratings from patients with stroke and therapists. Recommendations to enhance compliance are provided.

## Introduction

Stroke is a leading cause of acquired disability in adults worldwide, significantly impacting individuals’ quality of life due to persistent motor and cognitive impairments [1]. Importantly, these impairments in patients with stroke are often accompanied by other issues like fatigue and depressive symptoms, significantly impacting quality of life and independence [2]. Stroke is a significant contributor to cognitive decline and dementia, being the second most common cause after Alzheimer disease [2,3].

Rehabilitation strategies for individuals after a stroke primarily focus on restoring impaired motor control to enhance the independence of those affected [4]. An interdisciplinary approach that considers individual needs is essential for the success of rehabilitation and the overall quality of life for patients [5]. Thus, the advancement of options for improved, sustainable, and effective follow-up care in stroke rehabilitation is urgently needed [6]. Technological innovations can play an essential role in efforts to maintain the motor and cognitive abilities of affected individuals at the highest possible level. By integrating new technologies into rehabilitation programs, recovery outcomes can be enhanced, while early intervention and rehabilitation are crucial for improving outcomes and enhancing recovery after a stroke [7].

In addition to face-to-face rehabilitation, telerehabilitation has emerged due to advancing digitalization in the health care sector. It has been found that telerehabilitation in physiotherapy is comparable to in-person rehabilitation for conditions such as stroke [8,9]. In line with that, studies have shown that telerehabilitation can effectively deliver therapeutic interventions, maintain patient engagement, and achieve similar outcomes to traditional therapy, making it a valuable option for patients who may have difficulty attending in-person sessions [10].

Various mobile health apps have been developed for patients after a stroke, mostly focusing on upper extremity function, secondary prevention, or language skills [11]. Additionally, according to a review by Rintala et al [12], most studies investigating these apps focused on patients with chronic stroke, who are mildly impaired [13]. Although existing mobile health apps have shown positive effects on various stroke-related impairments, a substantial gap remains in the development of apps specifically targeting goal-oriented exercises and also integrating educational components [14].

The newly developed Blended Clinic platform (Blended Clinic AI GmbH) consists of a mobile app for patients (Blended Clinic, Version 1.0.1 [iOS]; 1.3 [Android]), a web-based portal for therapists, and a database. The system is designed to provide home-based exercise programs for different patient groups by integrating telerehabilitation with a blended care approach. Blended care itself refers to a health care approach that combines traditional in-person services with digital or remote care options. It aims to provide a more flexible and comprehensive approach to health care delivery, enabling diverse patient populations, including individuals after stroke in the subacute and chronic phases, to receive care through multiple modalities [15]. To date, an earlier iteration of a training program for patients after a stroke, in conjunction with a previous version of the Blended Clinic platform, has been evaluated for user interaction with the app and for acceptability among patients with chronic stroke [16]. It was found to be highly acceptable, with strong user engagement, good task adherence, and small clinical improvements in quality of life [16].

Based on these positive findings, the new Swiss Tele-Assisted Rehabilitation and Training (START) program was developed. It builds on the same core technology but extends the intervention and addresses the research gap in 2 ways: first, by further developing the content with a stronger focus on the needs of patients with moderate-to-severe impairments; and second, by evaluating its use in a new clinical context, the subacute phase of stroke rehabilitation, particularly the transition phase from inpatient to outpatient rehabilitation.

The aim of the present study was to describe the development process of the new START program and to assess the perceptions of both therapists and patients after a stroke regarding the usability and functionality of certain components of the START program, provided via the updated Blended Clinic platform, during test sessions.

## Methods

### Hardware and Software Components of the Blended Clinic Platform

The Blended Clinic platform consists of a web-based portal for therapists and a mobile app for patients. It is intended to be installed on the mobile devices of patients and comprises components for training, coaching, and monitoring. The content for these components is provided by the respective program modules, such as START, which was developed and tested within this project.

1. Training: in conjunction with a targeted training program, exercises can be performed daily, individually, and to the necessary and feasible extent. Through the app, patients are provided with a personal training plan tailored to their individual impairments and rehabilitation goals, including exercise videos. The exercise content can be adjusted to the achieved functional level.
2. Coaching: the app ensures that individual training is supported by a personal therapist to maintain high motivation levels. Patients’ questions about training and general topics related to stroke can be promptly addressed, both on-site and primarily through a digital chat function, with therapists available daily during working hours on weekdays.
3. Monitoring: the app also facilitates the collection of health-related, measurable parameters to support the ongoing individual optimization of the training plan. In this study, patient-reported outcome measures (PROMs), self-conducted blood pressure measurements, and activity tracking with a wrist-worn device (GENEActiv, Activinsights) were applied.

### The START Program

The main objective of START was to create a training program to be used with the Blended Clinic app specifically designed for patients with moderate to severe motor impairments during the early subacute stages of rehabilitation. We developed the START content in line with recent exercise recommendations [17-20] and stroke guidelines [21,22]. While these guidelines offer valuable recommendations, they do not specify explicit exercise instructions or dosages.

Three workshops were performed with a group of 7 clinicians, including 2 clinical physiotherapy neurological experts with over 25 years of professional experience and one research physiotherapist with a doctoral degree, 3 clinical physiotherapists with at least 3 years of neurological experience, and 1 sports scientist. The participating therapists served as clinical instructors in the clinic and, in part, as lecturers and researchers.

**Figure 1.**
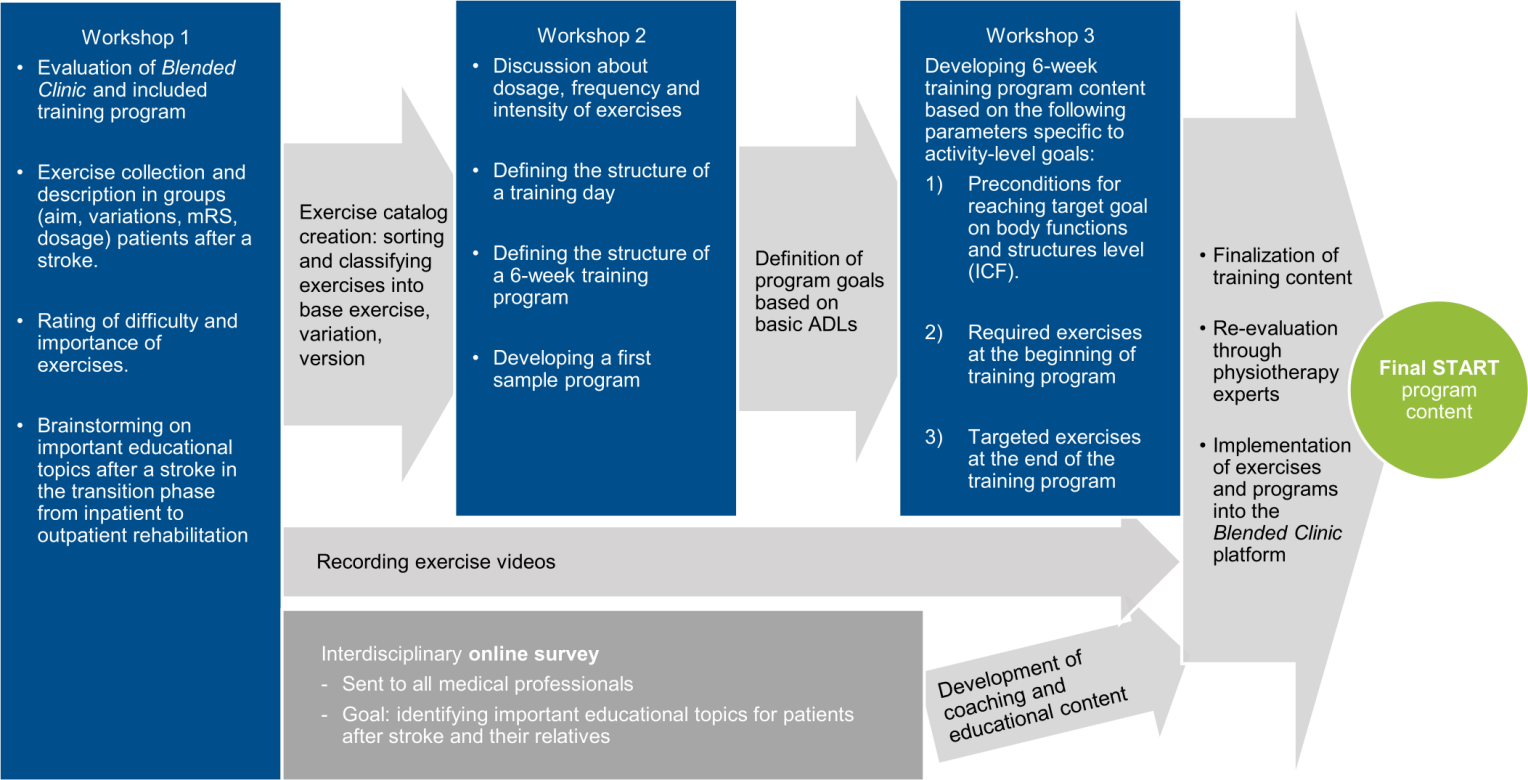
Development process of the Swiss Tele-Assisted Rehabilitation and Training (START) program content and the main focus of each workshop. ADL: activities of daily living; ICF: International Classification of Functioning, Disability and Health; mRS: modified Rankin Scale; START: Swiss Tele-Assisted Rehabilitation and Training

In these workshops, important exercises and exercise variations for patients after stroke were identified and classified into different disability levels based on the modified Rankin Scale (mRS) [23,24] scores 1-5 (Figure 1). Exercises were further categorized into lower extremity, trunk exercises, balance and coordination exercises, and breathing exercises. In total, this resulted in the creation of 224 new exercises, resulting in a pool of around 300 exercises. In addition, for the presentation within the Blended Clinic platform, an introductory text outlining the aim of each exercise and a video instruction demonstrating how to perform the exercise were developed. Please refer to the supporting information for further details regarding the exercises (Multimedia Appendix 1).

Patient exercises were compiled to create 10 6-week programs, each with an overarching goal. The activity goals of such a program were based on abilities that are necessary to perform activities of daily living (ADL). The experts identified key components necessary for establishing individualized program goals, such as the ability to transfer to the toilet. For instance, a target exercise could involve standing for at least 60 seconds while engaging in arm activities. For this, a preliminary exercise might consist of “standing independently for 30 seconds without any arm activity.”

Within each 6-week program, exercises progressed in terms of the number of sets, number of repetitions, and difficulty level. The exercise sessions, each lasting around 15 minutes and conducted daily, consisted of 3 phases, including a warm-up that typically involved a breathing or sensory stimulation exercise; a main component focusing on balance, strength, or endurance; and a cool-down that incorporated flexibility exercises. Additionally, care was taken to alternate strength, endurance, and balance training each day. Patients received information regarding training safety, content, meaning, and purpose of each exercise through the app.

In addition to the training programs, informational and educational materials for patients were developed. Relevant topics were identified by conducting an in-house interdisciplinary survey using the Findmind tool (Findmind). Based on the results from 90 health care professionals, several topics were identified, and content was developed. Podcasts were recorded covering the topics of fatigue, sleep, training, nutrition, and psychological well-being, along with the creation of 5 infographics. In addition to the regular motivational and informational messages sent via chat, this content constitutes an essential component of patient coaching and education and the blended care approach.

The START program was designed to be introduced in an inpatient rehabilitation setting and continue during the transition phase from inpatient to outpatient rehabilitation and then continue in the community. In the context of this usability study, not all the described content was used and investigated; rather, our focus was limited to an inpatient setting and specific components of START provided via the Blended Clinic platform.

### Evaluation of Usability and Functionality of START

#### Participants

The evaluation of the usability and functionality of the app-based training was conducted as a single-center study primarily with patients after a stroke in their subacute stage and secondarily with therapists. The study took place in a neurological rehabilitation clinic in northwestern Switzerland. We planned to include 10 patients and 10 therapists, starting in December 2023. Inpatients and outpatients attending the clinic’s day center were recruited by screening internal patient lists against the predefined eligibility criteria. The criteria comprised age ≥18 years, mRS 3-5, no severe aphasia, severe visual, neurological, cardiopulmonary, psychiatric, or orthopedic impairments that would prevent a patient from following the instructions, and no recent surgery or fractures. Therapists were included in the study if they held at least a bachelor’s degree in physiotherapy, occupational therapy, speech and language therapy, or sports and exercise science, and if they had at least one year of experience in stroke rehabilitation. Therapists were invited via email to participate in the study.

#### Procedures

**Figure 2.**
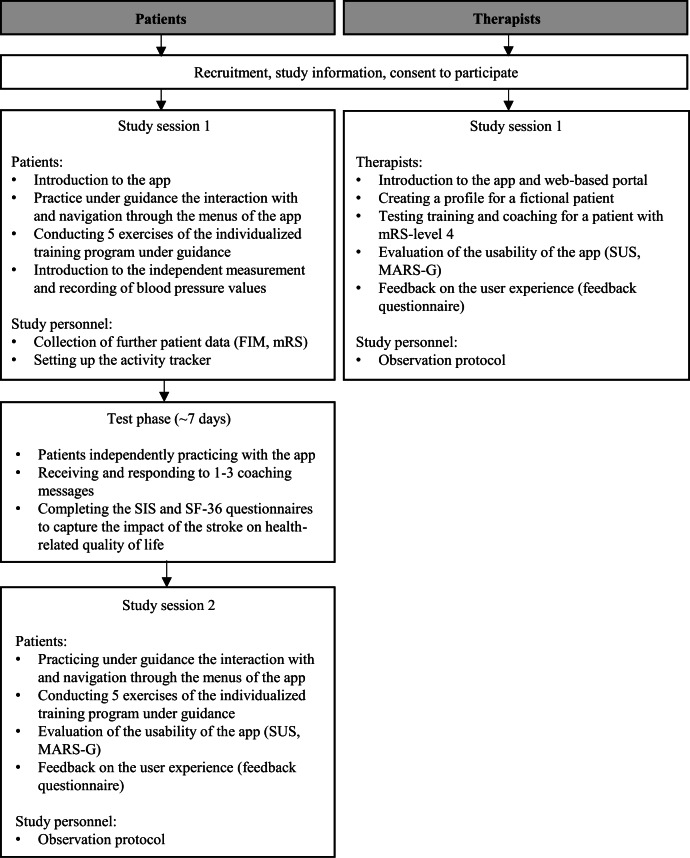
Study flowchart. FIM: Functional Independence Measure, MARS-G: Mobile App Rating Scale; mRS: modified Rankin Scale; SF-36: 36- Item Short Form Survey Instrument; SIS: Stroke Impact Scale; SUS: System Usability Scale.

For the course of the study, please refer to the study flow chart (Figure 2). As demonstrated here, the therapists participated in a single session, whereas patients underwent 2 sessions, with a one-week testing phase in between.

Patients had the opportunity to familiarize themselves with the app under supervision. During the introductory session with the study personnel, patients were instructed in the exercises assigned according to their mRS score and were trained in safe exercise execution. Besides, patients were also instructed on blood pressure measurements and on how to complete 2 questionnaires through the app (Stroke Impact Scale [SIS; 25,26], 36-Item Short Form Survey Instrument [SF-36; 27,28]). Patients subsequently tested the functionality of the app independently for a duration of one week. Before starting a daily exercise session, they received an automatic message reminding them to pay attention to their own safety during training. Furthermore, patients were provided with 2 information sheets, one outlining the criteria for discontinuing the exercises (eg, feeling unwell) and the other containing general information on the training environment (eg, ensuring that a table and chair were available for support). Patients wore an activity tracker (GENEActiv, Activinsights) for the week. During the second session following the testing phase, patients were asked to perform predefined tasks within the app under the guidance of the study personnel, for instance, to follow exercise instructions and to write a message to the coach. Patients were observed to document this process, to evaluate their performance, and thus the comprehensibility of the necessary interactive steps within the app.

Therapists were also introduced to the use of the app and had the opportunity to test it at the beginning of the session. They subsequently were instructed to perform specific tasks on the web-based dashboard platform, such as selecting and sending a questionnaire. Their performance was also observed and documented. Finally, the therapists themselves evaluated the usability of the system.

#### Outcome Measures

For the subsequent assessment of usability, the System Usability Scale (SUS) [29], the German version of Mobile App Rating Scale (MARS-G) [30-32] and a customized feedback questionnaire (Multimedia Appendix 2) were used.

The widely used SUS is a questionnaire that assesses the usability of a system through a 10-item survey. Each item is rated on a 5-point Likert scale, allowing users to express their level of agreement or disagreement with various statements about the system.

MARS-G assesses the multidimensional quality of mobile health apps and comprises 4 components, including engagement (Section A), functionality (Section B), esthetics (Section C), and information quality (Section D). Each domain is rated on a 5-point Likert scale, where 1 indicates “insufficient,” 2 denotes “poor,” 3 represents “acceptable,” 4 signifies “good,” and 5 reflects “excellent.” Additionally, the MARS-G includes 2 sections for the subjective evaluation of characteristics within the domains of subjective app quality (Section E) and perceived impact of the app (Section F). The German version of MARS-G that we used has been supplemented with a further section (Section T) that focuses on the therapeutic benefits associated with the app [32]. For all sections, the maximum achievable score is 5 points [32]. The therapists were asked to complete the entire MARS-G, while the patients were only provided with Sections A, B, C, D, E, and F for evaluation.

The training experiences with and the usability of the app were assessed using a self-constructed, standardized feedback questionnaire (Multimedia Appendix 2). This questionnaire used a 5-point scale and included specific questions regarding the app and was pilot tested with one patient. The feedback questionnaire aimed to provide deeper insights into the participants’ opinions about the training platform. For patients, the questionnaire contained 8 questions related to user experience, along with a section comprising 3 questions regarding the use of the blood pressure monitor, activity tracker, and smartphones or tablets. In contrast, therapists completed a questionnaire consisting of 9 questions about user experience.

During the study sessions for patients and therapists, the study personnel also completed an observation protocol (see Results for task details). This protocol facilitated the evaluation of the usability and user experience for both patients and therapists by an external observer, assessing the extent to which the app is useful and applicable, as well as identifying any existing issues. The study personnel also evaluated the emotional reactions by observation of the patients while they were navigating through the app and while performing the exercises, using the following categories (present and not present): attention, joy, curiosity, astonishment, boredom, nervousness, confusion, anger, and rejection.

### Data Analysis

The adherence of the participating patients to the START program protocol was derived from the execution of the planned training sessions, the number of blood pressure measurements, the completion of the questionnaires, and the duration of activity tracker usage. For all parameters, adherence was assessed in relation (ratio) to the intended use, with the following classifications defined: adherence of ≥75% of the planned activities was categorized as high adherence, 50%‐74% as moderate adherence, 25%‐49% as low adherence, and <25% as no adherence [33]. In this study, the tracker was used solely to assess whether the patients would tolerate wearing it, with a view to planning further studies and monitoring activity levels over a longer period.

- Training: number of completed training sessions versus planned training sessions. Patients received an exercise program consisting of 5 exercises each day. An adherent training day was defined as having performed at least 3 out of 5 exercises, quantified by spending a minimum of 20 seconds and a maximum of 5 minutes for each exercise on the app screen.
- Monitoring:
  - Blood pressure measurements: patients were instructed to conduct 3 blood pressure measurements each morning. Days on which the respective patient entered data themselves (eg, blood pressure) were classified as adherent.
  - Activity tracker: an adherent day was defined as a day on which the activity tracker was worn for a minimum of 5 hours on the first and last day or for at least 10 hours on each intervention day, consistent with established practices [34]. In this study, the focus was solely on understanding whether the trackers were accepted and worn, in preparation for a larger study.

All data were analyzed descriptively using JASP (version 0.18.0, The Jasp Team), R Studio (version 2023.06.0; Posit PBC) and Microsoft Excel (Excel 2019; Microsoft Corporation).

### Ethical Considerations

The study received approval from the independent ethics committee of Northwestern and Central Switzerland (EKNZ, reference number: 2023‐01783). All participants provided written informed consent and all participant data were de-identified. The participating therapists received compensation for participation of approximately $190.

## Results

### Overview

**Figure 3.**
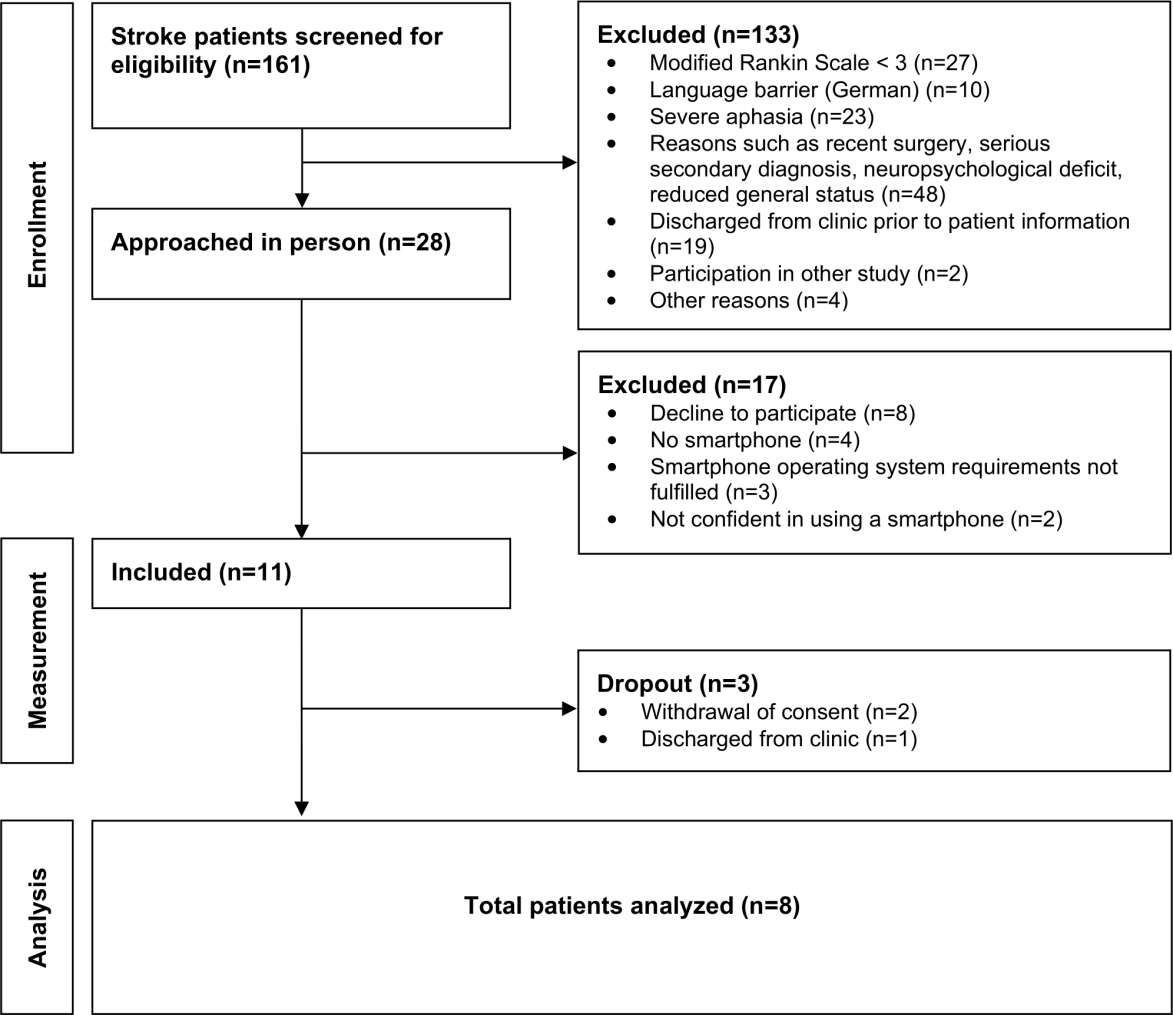
Study patient flowchart.

From December 2023 to March 2024, a total of 161 patients with stroke were screened for eligibility (Figure 3). Eight patients (6 females) completed the study. The patient group had a mean age of 61.5 (SD 12.7) years with an average duration since the stroke of 180 days (265 days, range: 15-618 days) and a Functional Independence Measure (FIM) [24,35] score of 93.8 (SD 12.8; see Table 1).

**Table 1.** Patient characteristics.

Patient demographics and clinical characteristics	Patient number							
	1	2	3	4	5	6	7	8
Sex	Female	Male	Female	Female	Female	Female	Male	Female
Age (years)	77	57	72	74	56	59	38	59
Stroke type	Ischemic	Ischemic	Ischemic	Ischemic	Ischemic	Hemorrhagic	Hemorrhagic	Ischemic
Time since stroke (days)	26	58	28	15	48	44	618	600
FIM^a^ total score (18-126)	96	103	119	90	84	90	91	77
FIM-motor (13-91)	65	75	86	64	59	65	68	—^b^
FIM-cognitive (5-35)	31	28	33	26	25	25	23	—
Modified Rankin Scale (0-5)	4	4	3	3	4	3	3	4
Smartphone operating system	iOS	iOS	Android	Android	iOS	Android	iOS	iOS

a FIM: Functional Independence Measure.

b Not available.

Ten therapists (8 females) with a mean age of 35.3 (SD 11.2) were enrolled. They were mostly physiotherapists (n=7) and occupational therapists (n=3).

### Outcome Measures

The patients rated the usability of the app, including training content and monitoring, with an average SUS score of 86.6 out of 100, which is slightly higher than the score of the therapists, who reported a score of 83.3 out of 100 (Tables2  3).

**Table 2.** Results from the System Usability Scale for all patients.

SUS score	Patient number								Mean (SD)	Median (IQR)
	1	2	3	4	5	6	7	8		
SUS^a^ (total score)	97.5	87.5	80	100	95	70	87.5	75	86.6 (10.9)	87.5 (20.6)
SUS^a^ (per item)										
Item 1	5	5	4	5	5	3	4	5	4.5 (0.8)	5 (1)
Item 2	2	2	2	1	1	3	1	5	2.1 (1.4)	2 (1.8)
Item 3	5	4	5	5	5	3	5	5	4.6 (0.7)	5 (0.8)
Item 4	1	1	3	1	1	1	1	1	1.3 (0.7)	1 (0)
Item 5	5	4	5	5	5	4	3	3	4.3 (0.9)	4.5 (1.8)
Item 6	1	1	2	1	1	3	1	5	1.9 (1.5)	1 (1.8)
Item 7	5	5	5	5	3	5	3	5	4.5 (0.9)	5 (1.5)
Item 8	1	2	1	1	1	1	1	1	1.1 (0.4)	1 (0)
Item 9	5	5	4	5	5	3	5	5	4.6 (0.7)	5 (0.8)
Item 10	1	2	3	1	1	2	1	1	1.5 (0.8)	1 (1)

a SUS: System Usability Scale.

**Table 3.** Results from the System Usability Scale (SUS) for all therapists.

SUS^a^ score	Therapist number										Mean (SD)	Median (IQR)
1	2	3	4	5	6	7	8	9	10
SUS^a^ (total score)	95	97.5	62.5	90	90	80	77.5	85	65	90	83.3 (12.0)	87.5 (16.9)
SUS^a^ (per item)												
Item 1	5	5	4	5	4	3	4	3	3	4	4.0 (0.8)	4 (2)
Item 2	1	1	2	1	1	1	2	1	1	1	1.2 (0.4)	1 (0.3)
Item 3	5	5	3	4	5	4	4	5	2	4	4.1 (0.9)	4 (1.3)
Item 4	1	1	1	1	1	1	1	1	3	1	1.2 (0.6)	1 (0)
Item 5	4	5	5	4	4	4	4	3	5	5	4.3 (0.7)	4 (1)
Item 6	1	1	4	1	2	2	1	1	1	2	1.6 (1.0)	1 (1)
Item 7	5	5	5	4	5	5	4	4	3	5	4.5 (0.7)	5 (1)
Item 8	1	1	3	1	1	2	2	1	1	1	1.4 (0.7)	1 (1)
Item 9	4	4	2	4	4	4	3	4	2	4	3.5 (0.8)	4 (4)
Item 10	1	1	4	1	1	2	2	1	3	1	1.7 (1.1)	1 (1)

a SUS: System Usability Scale.

**Figure 4.**
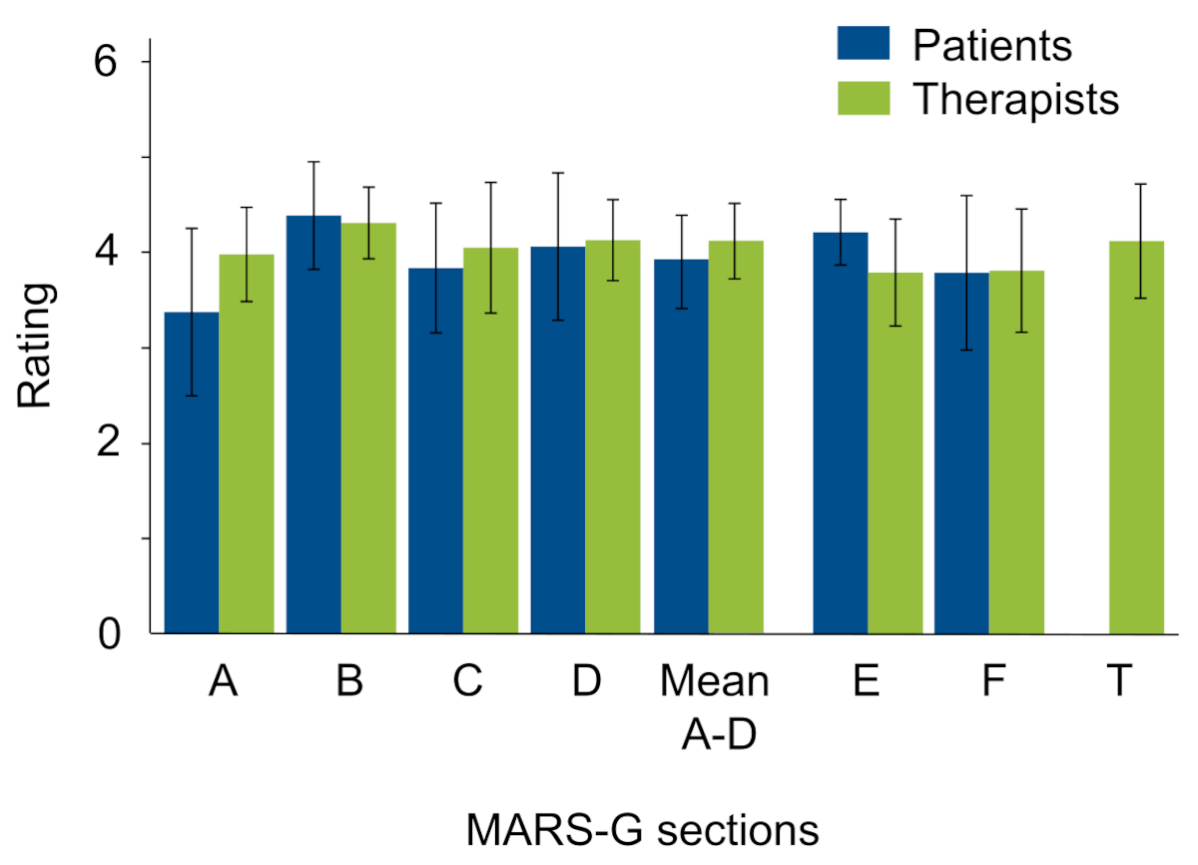
The German version of Mobile App Rating Scale (MARS-G) ratings of patients and therapists. A: engagement; B: functionality; C: esthetics; D information quality; E subjective quality; F: perceived impact; MARS-G: German version of Mobile App Rating Scale; T: therapeutic benefits.

For the MARS-G, both patients and therapists generally rated the user experience and usability of the app as acceptable to good, with the mean scores of Sections A-D being 3.9 for patients and 4.1 for therapists, as well as from the mean scores of the individual sections (Figure 4). The therapists rated no section below 4.0 points, thus categorizing all sections as good. Notably, they tended to rate the app slightly higher than the patients, particularly in the sections “Engagement” and “Aesthetics.” The section on “Functionality” received the highest ratings from patients and therapists. A table with the scores from patients and therapists is provided in the supporting information (Multimedia Appendix 3).

The results of the feedback questionnaire indicated a generally positive perception of the app and the devices. The patients had mixed opinions regarding the handling of the devices in terms of self-monitoring blood pressure and wearing the activity tracker. The latter was partly perceived as somewhat bothersome. The operation of the app on the tablet or smartphone was deemed to be relatively straightforward.

Among the therapists, there was a generally positive evaluation of the system. The question regarding the self-explanatory nature of the web-based dashboard for therapists received the lowest rating, while the question regarding technical functionality received the highest rating (Multimedia Appendix 4 for patients and therapists). Overall, all involved therapists expressed a desire to incorporate the app into their therapy planning.

### Observation Protocols

**Figure 5.**
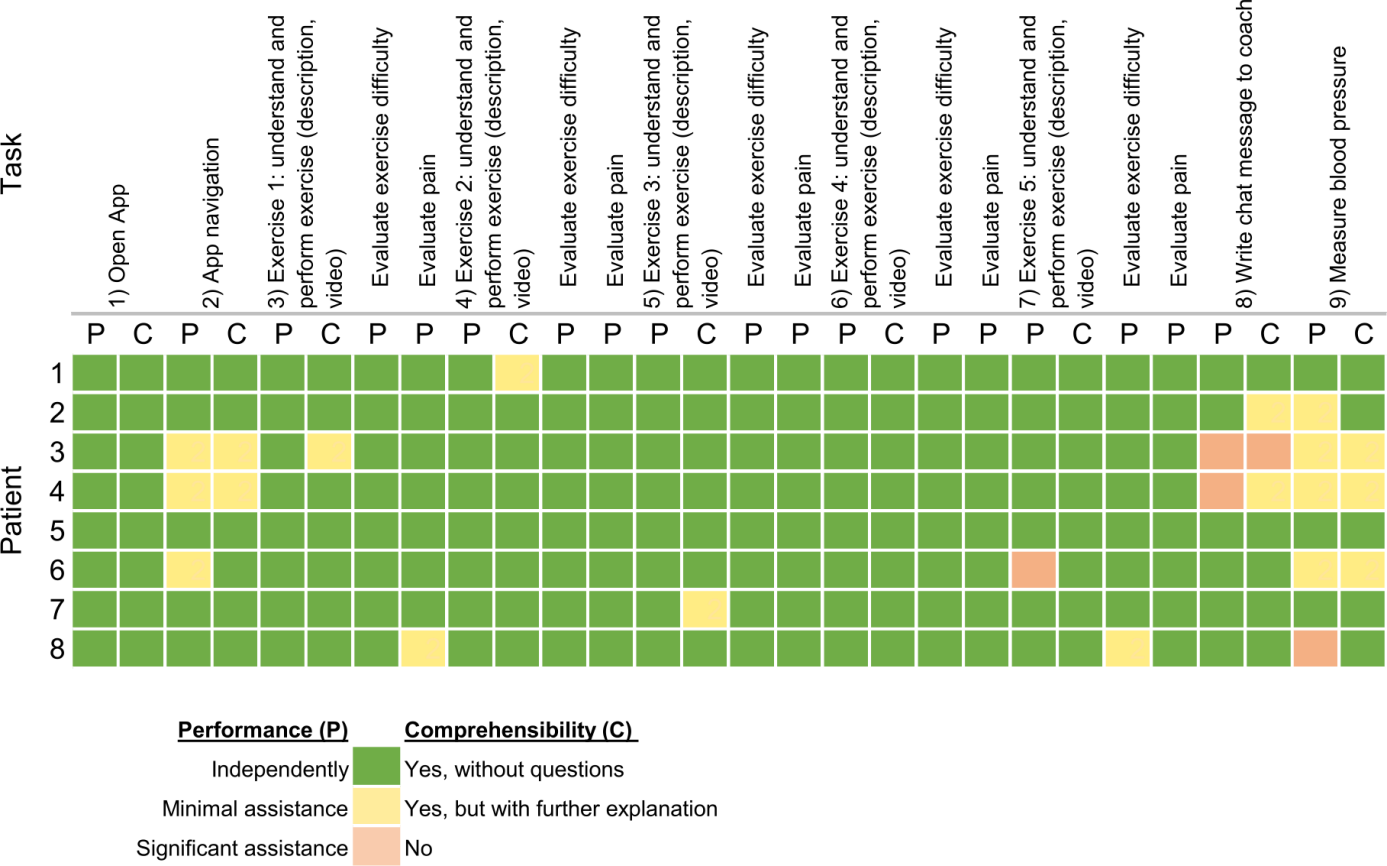
Evaluation of patients’ task performance.

Some patients encountered minor challenges in navigating the app’s menus. A few needed substantial assistance in using the chat function to communicate with the coach or had follow-up questions. Patients required the most assistance with blood pressure measurement, with the majority needing at least some help during the measurement process (Figure 5). The emotions of attention, joy, curiosity, and astonishment were repeatedly mentioned. Except for one individual, all patients were engaged and attentive. Two-thirds of the patients exhibited joy throughout the observation. Curiosity and amazement were also noted in approximately half of the patients. In contrast, emotions such as rejection, anger, confusion, and nervousness were rarely observed, with 2 patients exhibiting these feelings.

**Figure 6.**
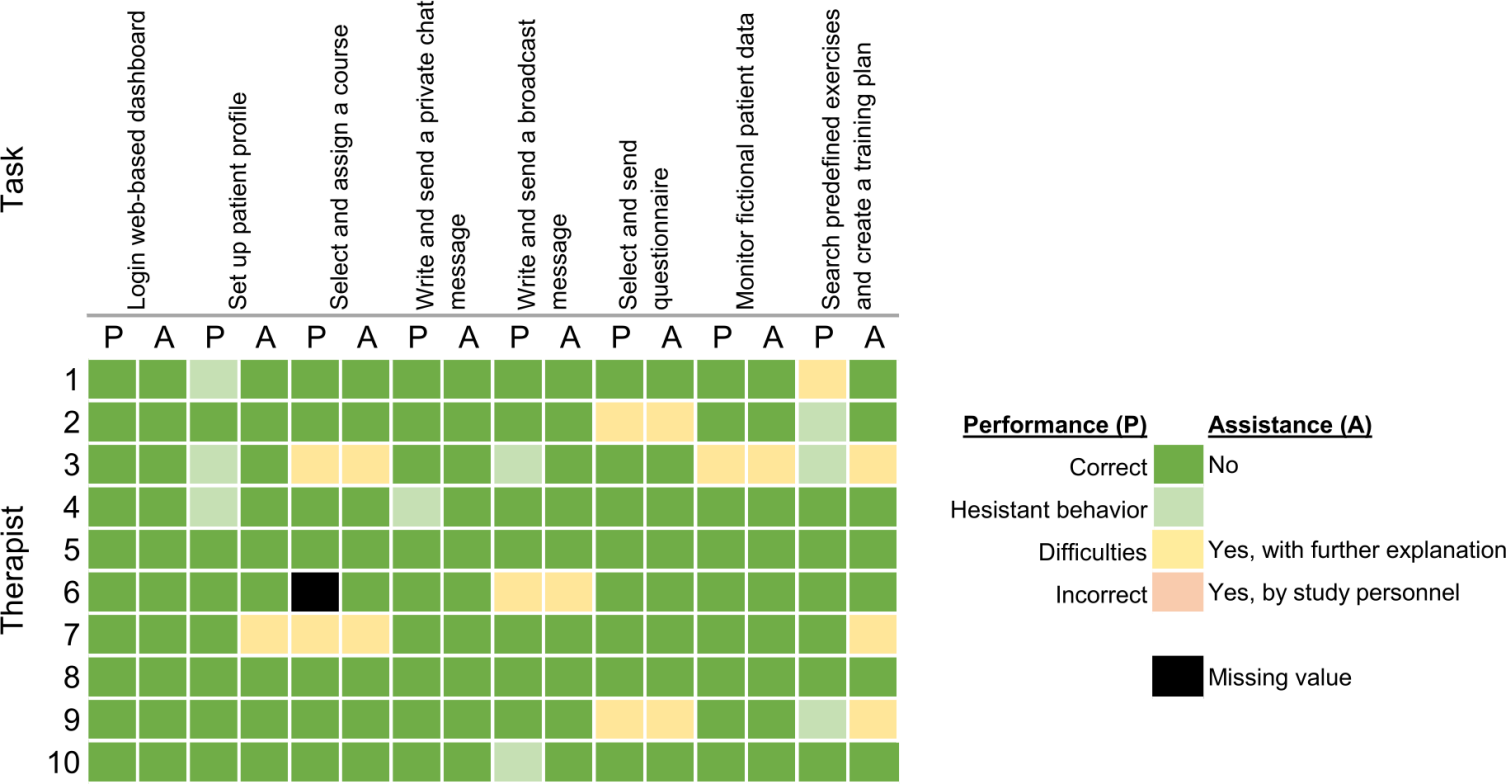
Evaluation of therapists’ task performance.

All therapists were able to perform the assigned tasks. Only a few needed some further explanations (Figure 6).

### Adherence to the START Program Protocol

Overall, patients’ adherence to the protocol varied and was moderate for the test phase between study sessions. Adherence regarding training and monitoring differed distinctively among individual patients (Multimedia Appendix 5). Some patients consistently wore the activity tracker but did not adhere to the planned exercises or blood pressure measurements. There were no adverse events reported during the one-week trial phase.

- Training: adherence to training was low on average. Only one patient trained on most days (≥75% of planned training sessions), while 3 patients showed moderate adherence (between 50% and 74% of the planned sessions). One patient revealed low adherence to the training plan (between 25% and 49%), and 3 had no adherence (<25%). Two patients could not perform exercises on one day or 2 days due to technical problems. Furthermore, some patients skipped part of or all exercises in the app, as noticed by the short time spent on the exercise screens.
- Monitoring:

- Blood pressure measurements: only one patient performed the blood pressure measurement every day. Three patients measured blood pressure on more than half of the planned days.
- Activity tracker: most patients (n=6) showed high adherence (≥75% of planned days) wearing the activity tracker. The adherence of one patient was low (25%‐49%), and one patient did not wear the tracker at all. In the case of this latter patient, however, the reason was a skin allergic reaction. There was no reason for the patients to remove the tracker, whether for charging, sleeping, or bathing, which may have contributed to the generally high adherence regarding the wearing of the trackers.

## Discussion

### Principal Findings

The aim of this study was to introduce our newly developed START program and initially determine how patients after a stroke and therapists evaluate the usability and functionality of START within the Blended Clinic platform. The following key findings were identified:

- This study demonstrated that both patients and therapists consider the Blended Clinic platform, including the START program, to be user-friendly.
- Regarding the usability of the app for patients after a stroke, the study yielded generally positive results. However, there remains potential for improvement to enhance long-term compliance with the program by patients and to ensure ease of use for therapists in the future.

### Usability Evaluation

Evaluating usability is crucial to engage both patients and therapists early in the development process and to incorporate their valuable feedback into ongoing product refinement [36]. The initial insights into the usability of the telerehabilitation platform Blended Clinic in combination with the START program demonstrated good user-friendliness. Most of the given tasks were completed by the patients without assistance, which reassures us that the intended independent use of the Blended Clinic app is achievable. This is supported by the high SUS scores (83.3 among therapists and 87.2 among the participating patients with stroke), indicating the Blended Clinic platform with the START program as user-friendly. In a meta-analysis of digital health apps, the benchmark was a mean score of 68 with an SD of 12.5 [37], which is considerably exceeded in this study.

The evaluation based on the MARS-G allows for the assessment of the overall quality of the app-based training program, as well as the identification of both strengths and weaknesses through the mean scores of the individual sections [30]. In general, both therapists and patients rated the user experience and usability of the app as acceptable to good. Notably, therapists tended to rate the app slightly higher than the patients, particularly in the sections on “Engagement” and “Aesthetics.” Patients appear to be somewhat more critical in their evaluation of the app. This discrepancy may be attributed to the differing requirements and expectations of the 2 groups regarding the app [38].

Although the generalizability and credibility of the results from the feedback questionnaire are limited, it provided valuable insights, for example, concerning the handling of the blood pressure monitor, which can be applied in future studies. The results of the feedback questionnaire also indicated a generally positive perception of the app and the devices by the patients, although several areas for potential improvement have been identified. The technical functionality was regarded as having development potential. However, technical problems are quite common in studies investigating mobile health apps, as described in a scoping review on problems and barriers related to the use of these apps [39].

The therapists’ feedback additionally provided valuable insights into the practical implementation of the app. The therapists expressed a consistently positive response to the question of whether they would like to use the system in their therapy. The ratings indicated a high level of acceptance, overall satisfaction, and the potential of the system as a telerehabilitation option during the rehabilitation process. Our results are consistent with previous research. Physiotherapists are generally open to digital health technologies but frequently emphasize barriers to their implementation. In the study by Martinsen et al [37], therapists expressed concerns particularly about technical aspects, reimbursement issues, and establishing and maintaining a therapeutic relationship with patients [40]. The Blended Clinic platform with the START program is intended to address these issues through a blended care approach, which combines digital elements and personal interaction. The therapists in our study highlighted other important considerations, such as integration into existing therapy workflows, the need for flexibility in scheduling, and potential barriers related to staffing or digital infrastructure.

### Patient Adherence to Protocol

The results of the one-week test phase indicated low adherence to the START training exercises among patients, despite the exercises being designed to be manageable and requiring only 10 minutes of daily commitment. Several factors may have contributed to the low adherence rates.

Participation in the study was conducted alongside the patients’ regular therapy regimen, which may have impeded their motivation to fully adhere to the study protocol. In addition, the exercises were rather easy for some patients, which might have influenced their adherence. Exercise difficulty and progression should be evaluated in further investigations.

While the presence of instructional videos and access to a physiotherapist via chat provided valuable resources, patients may still have struggled with their training. It is important to consider that impaired cognitive abilities can hinder protocol adherence. A study involving patients with stroke showed that factors such as age and disarticulation significantly influence rehabilitation exercise adherence [41]. From our perspective, it would be beneficial to assess and individualize the amount and method of external support and motivation required. Further strategies may have been needed to enhance patient engagement and adherence to the exercise program, for example, family involvement.

Regarding low adherence to blood pressure measurements, patients may have perceived little added value in measuring their blood pressure themselves since these were routinely taken by the care personnel during inpatient stay. Not all patients were equally affected after a stroke, and some may have had further difficulties using the device due to motor function limitations, uncontrolled movements, or even hemiplegia, which could explain the issues encountered during blood pressure measurement.

On the contrary, the adherence to wearing the activity tracker was quite high. Such trackers have high potential to deliver meaningful data for the health care professionals, for example, step counts or time spent on specific activity levels. This activity tracker data could also be included directly in the Blended Clinic platform to increase the motivation and physical activity of patients. Such an approach is described in the pilot study by Paul et al [39], where a gamified approach was used to enhance step count goals [42].

Grau-Pellicer et al [40] conducted a randomized controlled trial using a similar program in conjunction with a smartphone app [43]. The authors could show positive results, for example, an improved activity level in the intervention group, but there was a low adherence to app use. They stressed the importance of developing technologies adapted to patients with stroke that are easy to use in order to increase adherence [43]. In our study, we tested only part of the START program, a one-week period, with a selected patient group (mRS 3‐5), as usability was of particular importance to us at this stage. In contrast, adherence rates are generally high (≥80%) in telerehabilitation studies for patients with stroke, although this aspect is often underreported [44].

However, a randomized controlled trial by Emmerson et al [42] found no superiority of a home-based exercise program delivered via electronic tablet compared to a paper-based version, underscoring the importance of remote therapeutic support [12]. Consistent with this, promising results have been reported when remote support was provided to enhance adherence to home exercises in individuals with musculoskeletal conditions [45].

Usability evaluations for a wide range of mobile health apps have been conducted across various target populations [46], including patients after a stroke [47], demonstrating beneficial effects for patients. In physical stroke rehabilitation, similar mobile apps were developed and evaluated, for example, ARMStrokes (Towson University) for upper extremity training [48], an app to increase physical activity [42], or a program involving caregivers [49]. These and other technologies show promising results, especially for improving upper extremity function [47].

However, despite the already available apps for patients after stroke, most apps have been developed outside Europe [11]. Furthermore, additional equipment, for example, a balance disc [50] or a virtual reality system [51], is often necessary. With START, we focused on individualized physiotherapeutic exercises that require no special equipment. Notably, our usability study deliberately included patients with moderate to severe disabilities—a group often excluded from similar research—thereby providing valuable insights into the applicability and accessibility of the intervention for a broader spectrum of stroke survivors.

### Limitations

Our usability study has several limitations that should be considered when interpreting the results. The sample size was small; however, we could include the planned patients after a stroke and therapists to evaluate the START program.

Furthermore, the intervention period for patients was only one week, which may not have been sufficient to draw conclusions about long-term adherence for the holistic START program. Extended usage periods are warranted to gain a deeper understanding of the factors influencing adherence to digital home-based exercise programs.

We also lack more detailed information about the patients, such as cognitive function or digital literacy. In our study, patients and therapists were asked to engage with a technical system and evaluate its usability and user experience. For study participation, patients were required to own a smartphone or tablet. Therefore, we assume that patients possess at least basic technical knowledge and feel confident in their ability to operate the technology. On the other hand, these requirements proved to be a challenge, as we experienced in a prolonged recruitment phase. However, the proportion of older individuals who own a smartphone is expected to rise [52], ensuring that access to mobile apps will be available for the majority of patients after a stroke. It can therefore be assumed that in the future, a significant proportion of patients after stroke will possess the necessary prerequisites to use these digital opportunities.

The feedback collected from patients and therapists was quite positive, but we cannot exclude the possibility of socially desired responses when interpreting the subjective outcomes. Ried et al [51] propose 8 preventive measures to address this issue; one of them is indirect questioning. Future studies should consider such measures to minimize this bias.

These limitations underscore the need for further research. Based on the findings from our study, we have designed a feasibility study aimed at a more comprehensive evaluation of the entire START program with a larger sample size. This evaluation will encompass the transition between inpatient and outpatient care, as well as the holistic content of the program covering all mRS levels. Additionally, we have established other focal points concerning the outcome measures, such as performance-based outcome measures.

### Conclusions

In summary, the Blended Clinic platform, including the START program, was initially considered user-friendly, with good usability ratings from both patients with stroke and therapists. The available data provide an insight into patient adherence and the underlying challenges. Future efforts should focus on optimizing technical functionality, enhancing user motivation, and integrating training into the daily routines of patients to potentially improve adherence. This study’s results and adjustments aimed at enhancing the motivational component and paving the way for a long-term evaluation within a multiweek clinical study, in which the transition between hospital stay and the use of the system in a home setting will be tested.

## Supplementary material

10.2196/77090 Multimedia Appendix 1 Exercise catalog.

10.2196/77090 Multimedia Appendix 2 Feedback questionnaires.

10.2196/77090 Multimedia Appendix 3 Mobile App Rating Scale (MARS-G) results.

10.2196/77090 Multimedia Appendix 4 Feedback questionnaire results of patients and therapists.

10.2196/77090 Multimedia Appendix 5 Adherence metrics for patients.

## Abbreviations

ADL: activities of daily living
MARS-G: German version of Mobile App Rating Scale
mRS: modified Rankin Scale
SF36: 36-Item Short Form Survey Instrument
SIS: Stroke Impact Scale
START: Swiss Tele-Assisted Rehabilitation and Training program
SUS: System Usability Scale
