# Supplementary material for: Telerehabilitation Following Stroke: Development of Training Content and Evaluation of an App-Based Training Program
Source: JMIR Rehabil Assist Technol. 2026 Mar 31;13:e77090. doi: 10.2196/77090 (PMC13037760; doi:10.2196/77090)
Supplement: Multimedia Appendix 1 [file rehab-v13-e77090-s001.pdf]

Exercise category	Exercise	Version	Variation	Difficulty level (based on mRS)					Exercise focus							
				1	2	3	4	5	strength	endurance	balance	motor/ movement	sensory stimulation	flexibility	warm-up/ cool-down	task relearning
lower extremity	posterior chain - plantarflexors	calf-raises	standard	x	x				x		x					
			beside wall	x	x	x			x							
			wall-support		x	x	x		x							
			one leg wall-support	x	x				x							
			one leg	x					x		x					
		plantarflexion activation	sitting			x	x	x	x			x			x	
			step position			x	x		x			x		x		
			tip toe hops	x					x		x	x				
balance	standing (static)	with support (table)	standard				x	x	x		x					x
			support-release			x	x		x		x					
			eye movements			x	x		x		x		x			
			head movements (right-left)			x	x		x		x					
			head movements (up-down)			x	x		x		x					
			pelvis lean			x	x	x	x		x					
			arm activity			x	x		x		x					
			household activities			x	x		x	x	x					x
		standard	standard			x	x		x		x					
			barefoot		x	x					x		x			
			eyes closed	x	x	x					x		x			
		closed feet	standard		x	x					x					
			barefoot		x	x					x		x			
			support-release			x					x					
			eyes closed	x	x						x		x			
core	motor control	supine	pelvic tilt	x	x	x	x	x				x			x	
			hip hike	x	x	x	x	x				x			x	
			lower trunk rotation	x	x	x	x	x				x			x	
		sitting	pelvic tilt	x	x	x	x	x				x			x	
			forward bend			x	x					x			x	
			trunk rotation		x	x	x					x			x	
		standing	pelvic tilt	x	x	x						x			x	
			pelvic rotation (wall)	x	x	x						x			x	
			trunk rotation	x	x	x						x			x	

Table 1 . Selected exercise examples from the START exercise catalog, including category, version and variations, mapped to difficulty level (based on modified Rankin Scale [mRS] grades 1–5) and primary exercise focus domains.
