# Supplementary material for: Telerehabilitation Following Stroke: Development of Training Content and Evaluation of an App-Based Training Program
Source: JMIR Rehabil Assist Technol. 2026 Mar 31;13:e77090. doi: 10.2196/77090 (PMC13037760; doi:10.2196/77090)
Supplement: Multimedia Appendix 2 [file rehab-v13-e77090-s002.pdf]

Table 1. Patient and therapist feedback questionnaires (German originals and non-validated English translations) assessing user experience, handling/usability, and perceived suitability and implementation factors for the system in stroke rehabilitation.

1) **Patients**

German version

Bewerten Sie folgende Fragen auf einer Skala von 1 bis 5.		
**Teil 1: Benutzererfahrung**		
1	Wie zufrieden waren Sie mit der Nutzung der Applikation?	☐ 1 (überhaupt nicht zufrieden)<br>☐ 2<br>☐ 3 (weder zufrieden noch unzufrieden)<br>☐ 4<br>☐ 5 (voll und ganz zufrieden)
2	Hat Ihnen das Training mit der Applikation Freude gemacht?	☐ 1 (überhaupt keine Freude)<br>☐ 2<br>☐ 3 (mittelmässig)<br>☐ 4<br>☐ 5 (sehr viel Freude)
3	War das Design der Applikation gut gewählt/ansprechend?	☐ 1 (überhaupt nicht)<br>☐ 2<br>☐ 3 (mittelmässig)<br>☐ 4<br>☐ 5 (sehr)
4	Würden Sie in Ihrer Therapie gerne mit dieser Applikation trainieren?	☐ 1 (überhaupt nicht gerne)<br>☐ 2<br>☐ 3 (mittelmässig)<br>☐ 4<br>☐ 5 (sehr gerne)
5	Haben Sie sich beim Training sicher geleitet gefühlt?	☐ 1 (überhaupt nicht)<br>☐ 2<br>☐ 3 (mittelmässig)<br>☐ 4<br>☐ 5 (sehr sicher)
6	Hat die Applikation technisch gut funktioniert?	☐ 1 (überhaupt nicht)<br>☐ 2<br>☐ 3 (mittelmässig)<br>☐ 4<br>☐ 5 (einwandfrei)
7	Waren Sie durch die Applikation motivierter zum Trainieren?	☐ 1 (unmotivierter)<br>☐ 2<br>☐ 3 (gleich motiviert)<br>☐ 4<br>☐ 5 (deutlich motivierter)
8	Für wie geeignet halten Sie die App für Patientinnen und Patienten nach Schlaganfall?	☐ 1 (überhaupt nicht)<br>☐ 2<br>☐ 3 (mittelmässig)<br>☐ 4<br>☐ 5 (sehr geeignet)

**Teil 2: Umgang mit dem Gerät**		
1	Wie sind Sie mit dem selbstständigen Blutdruckmessen zurecht gekommen?	☐ 1 (überhaupt nicht, nur mit Hilfe)<br>☐ 2<br>☐ 3 (manchmal alleine, manchmal mit Hilfe)<br>☐ 4<br>☐ 5 (sehr gut alleine)
2	Wie herausfordernd war das selbstständige Messen des Blutdrucks für Sie?	☐ 1 (sehr mühsam)<br>☐ 2<br>☐ 3 (ok)<br>☐ 4<br>☐ 5 (sehr einfach)
3	Wie oft haben Sie den Aktivitätsmesser getragen?	☐ 1 (fast nie)<br>☐ 2<br>☐ 3 (die Hälfte der Tage)<br>☐ 4<br>☐ 5 (jeden Tag)
4	Hat Sie das Tragen des Aktivitätsmessers gestört?	☐ 1 (ja, sehr)<br>☐ 2<br>☐ 3 (mittelmässig)<br>☐ 4<br>☐ 5 (überhaupt nicht)
5	Wie schwer war für Sie die Bedienung des Tablets/Smartphones?	☐ 1 (sehr schwer)<br>☐ 2<br>☐ 3 (mittelmässig)<br>☐ 4<br>☐ 5 (sehr einfach)

English version

Please rate the following questions on a scale from 1 to 5.		
**Part 1: User experience**		
1	How satisfied were you with the use of the system?	☐ 1 (not at all)<br>☐ 2<br>☐ 3 (moderately)<br>☐ 4<br>☐ 5 (very much)
2	Did you enjoy using the system?	☐ 1 (not at all)<br>☐ 2<br>☐ 3 (moderately)<br>☐ 4<br>☐ 5 (very much)
3	Was the design of the application well-chosen/ appealing?	☐ 1 (not at all)<br>☐ 2<br>☐ 3 (moderately)<br>☐ 4<br>☐ 5 (very much)
4	Would you like to use this application in your therapy?	☐ 1 (not at all)<br>☐ 2

		☐ 3 (moderately)<br>☐ 4<br>☐ 5 (very much)
5	Did you feel well-supported during the training?	☐ 1 (not at all)<br>☐ 2<br>☐ 3 (moderately)<br>☐ 4<br>☐ 5 (very much)
6	Was the technical performance of the application satisfactory?	☐ 1 (not at all)<br>☐ 2<br>☐ 3 (moderately)<br>☐ 4<br>☐ 5 (very much)
7	Have you been more motivated to train using the application?	☐ 1 (less motivated)<br>☐ 2<br>☐ 3 (moderately)<br>☐ 4<br>☐ 5 (much more motivated)
8	How suitable do you think the app is for patients after a stroke?	☐ 1 (not at all)<br>☐ 2<br>☐ 3 (moderately)<br>☐ 4<br>☐ 5 (very much)
**Part 2: Handling**		
1	How did you manage to measure your blood pressure independently?	☐ 1 (not at all, only with help)<br>☐ 2<br>☐ 3 (sometimes alone, sometimes with help)<br>☐ 4<br>☐ 5 (very well)
2	How challenging was measuring your blood pressure independently?	☐ 1 (very difficult)<br>☐ 2<br>☐ 3 (okay)<br>☐ 4<br>☐ 5 (very easy)
3	How often did you wear the activity tracker?	☐ 1 (almost never)<br>☐ 2<br>☐ 3 (half of the days)<br>☐ 4<br>☐ 5 (every day)
4	Did you find wearing the activity tracker bothersome?	☐ 1 (yes, very much)<br>☐ 2<br>☐ 3 (moderately)<br>☐ 4<br>☐ 5 (not at all)

2) Therapists

German version

Bewerten Sie folgenden Fragen auf einer Skala von 1 bis 5.		
1	Wie zufrieden waren Sie mit der Nutzung des Systems?	☐ 1 (überhaupt nicht zufrieden)<br>☐ 2<br>☐ 3 (weder zufrieden noch unzufrieden)<br>☐ 4<br>☐ 5 (voll und ganz zufrieden)
2	Hat Ihnen die Nutzung des Systems Freude gemacht?	☐ 1 (überhaupt keine Freude)<br>☐ 2<br>☐ 3 (mittelmässig)<br>☐ 4<br>☐ 5 (sehr viel Freude)
3	War der Inhalt des webbasierten Dashboards gut gewählt?	☐ 1 (überhaupt nicht)<br>☐ 2<br>☐ 3 (mittelmässig)<br>☐ 4<br>☐ 5 (sehr)
4	Waren Aspekte des Grafikdesigns (z.B. Schrift, Sprache, Grösse) des webbasierten Dashboards gut gewählt/ansprechend?	☐ 1 (überhaupt nicht)<br>☐ 2<br>☐ 3 (mittelmässig)<br>☐ 4<br>☐ 5 (sehr)
5	War das webbasierte Dashboard selbsterklärend, wussten Sie was Sie machen mussten?	☐ 1 (überhaupt nicht)<br>☐ 2<br>☐ 3 (mittelmässig)<br>☐ 4<br>☐ 5 (immer/sehr)
6	Hat das System technisch gut funktioniert?	☐ 1 (überhaupt nicht)<br>☐ 2<br>☐ 3 (mittelmässig)<br>☐ 4<br>☐ 5 (einwandfrei)
7	Würden Sie die *Blended Clinic* Applikation gerne in Ihre Therapie miteinbeziehen?	☐ 1 (überhaupt nicht gerne)<br>☐ 2<br>☐ 3 (mittelmässig)<br>☐ 4<br>☐ 5 (sehr gerne)

English version

Please rate the following questions on a scale from 1 to 5.		
1	How satisfied were you with the use of the system?	☐ 1 (not at all)<br>☐ 2<br>☐ 3 (moderately)<br>☐ 4<br>☐ 5 (very much)

2	Did you enjoy using the system?	☐ 1 (not at all)<br>☐ 2<br>☐ 3 (moderately)<br>☐ 4<br>☐ 5 (very much)
3	Was the content of the web-based dashboard well-chosen?	☐ 1 (not at all)<br>☐ 2<br>☐ 3 (moderately)<br>☐ 4<br>☐ 5 (very well)
4	Were aspects of the graphic design (e.g., font, language, size) of the web-based dashboard well-chosen/appealing?	☐ 1 (not at all)<br>☐ 2<br>☐ 3 (moderately)<br>☐ 4<br>☐ 5 (very well)
5	Was the web-based dashboard self-explanatory, did you know what you had to do?	☐ 1 (not at all)<br>☐ 2<br>☐ 3 (moderate)<br>☐ 4<br>☐ 5 (always/ very much)
6	Did the system function well technically?	☐ 1 (not at all)<br>☐ 2<br>☐ 3 (moderately)<br>☐ 4<br>☐ 5 (very well)
7	Would you like to include the *Blended Clinic* application in your therapy?	☐ 1 (not at all)<br>☐ 2<br>☐ 3 (moderately)<br>☐ 4<br>☐ 5 (very much)
8	In your opinion, what would be the benefits of using this system (app and web-based dashboard) in daily clinical practice?<br>*(Please list possible advantages.)*	
9	In your opinion, what could be potential barriers or obstacles to using such a system (application and web-based dashboard) in daily clinical practice? *(Please list possible barriers.)*	
