# Supplementary material for: Telerehabilitation Following Stroke: Development of Training Content and Evaluation of an App-Based Training Program
Source: JMIR Rehabil Assist Technol. 2026 Mar 31;13:e77090. doi: 10.2196/77090 (PMC13037760; doi:10.2196/77090)
Supplement: Multimedia Appendix 4 [file rehab-v13-e77090-s004.pdf]

Table 1. Patient and therapist ratings from the feedback questionnaire, presenting individual item scores and group summaries (mean ± SD and median [IQR]) for patient domains (user experience and handling) and therapist items.

	Patient No.									Md
**Item**	**1**	**2**	**3**	**4**	**5**	**6**	**7**	**8**	**M (SD)**	**(IQR)**
**Part 1: User experience**										
**1**	5	4	5	4	4	3	5	5	4.4 (0.7)	4.5 (1)
**2**	5	4	3	5	5	3	4	3	4.0 (0.9)	4 (2)
**3**	5	3	4	5	5	4	4	3	4.1 (0.8)	4 (1.8)
**4**	5	3	3	5	5	3	4	1	3.6 (1.4)	3.5 (2)
**5**	5	4	5	5	5	4	5	3	4.5 (0.8)	5 (1)
**6**	3	3	5	3	3	3	4	5	3.6 (0.9)	3 (1.3)
**7**	5	4	5	4	5	2	3	3	3.9 (1.1)	4 (2)
**8**	5	5	4	4	5	5	5	4	4.6 (0.5)	5 (1)
**Part 2: Handling**										
**1**	5	4	3	4	5	1	5	1	3.5 (1.6)	4 (3.5)
**2**	5	3	3	5	5	1	5	1	3.5 (1.7)	4 (3.5)
**3**	5	3	1	5	5	5	4	3	3.9 (1.4)	4.5 (2)
**4**	4	3	1	5	1	5	5	5	3.6 (1.7)	4.5 (3.5)
**5**	5	3	5	3	5	3	5	5	4.3 (1.0)	5 (2)

Md: Median; M: Mean; IQR: Interquartile range; SD: standard deviation;

	Therapist No.											Md
**Item**	**1**	**2**	**3**	**4**	**5**	**6**	**7**	**8**	**9**	**10**	**M (SD)**	**(IQR)**
**1**	5	5	4	4	4	4	4	3	3	5	4.1 (0.7)	4 (1)
**2**	5	5	4	5	5	3	3	3	4	4	4.1 (0.8)	4 (2)
**3**	5	5	4	5	4	4	4	3	4	5	4.3 (0.6)	4 (1)
**4**	5	5	3	5	4	3	4	4	3	4	4 (0.8)	4 (1.8)
**5**	4	5	2	4	5	3	3	5	3	3	3.7 (1.0)	3 (1.8)
**6**	5	5	5	5	4	4	5	5	4	4	4.6 (0.5)	4.5 (1)
**7**	5	5	5	5	4	4	4	4	4	5	4.5 (0.5)	4 (1)

Md: Median; M: Mean; IQR: Interquartile range; SD: standard deviation;
