# Supplementary material for: Telerehabilitation Following Stroke: Development of Training Content and Evaluation of an App-Based Training Program
Source: JMIR Rehabil Assist Technol. 2026 Mar 31;13:e77090. doi: 10.2196/77090 (PMC13037760; doi:10.2196/77090)
Supplement: Multimedia Appendix 5 [file rehab-v13-e77090-s005.pdf]

Table 1. Adherence metrics for each patient, showing wear time of the activity tracker, completion of scheduled blood pressure measurements, and completion of planned training exercises expressed as percentages of the intended total.

Patient	Activity tracker worn as a percentage of the total duration	Blood pressure measurements as a percentage of the planned measurements	Training: Percentage of the completed exercises relative to the planned total number.
1	75.0	66.7	50.0
2	37.5	33.3	50.0
3	0.0	20.0	80.0
4	85.7	20.0	20.0
5	85.7	40.0	40.0
6	100.0	50.0	16.7
7	100.0	100.0	16.7
8	75.0	66.7	66.7

Please note that one patient did not wear the tracker due to an allergic reaction to the material of the wristband.
