# Supplementary material for: Telerehabilitation Following Stroke: Development of Training Content and Evaluation of an App-Based Training Program
Source: JMIR Rehabil Assist Technol. 2026 Mar 31;13:e77090. doi: 10.2196/77090 (PMC13037760; doi:10.2196/77090)
Supplement: Multimedia Appendix 3 [file rehab-v13-e77090-s003.pdf]

Table 1. Mobile Application Rating Scale–German (MARS-G) results for patients and therapists, showing individual participant scores and group summaries (mean ± SD and median [IQR]) across MARS sections and overall app quality.

Dimension	Patient No.								M (SD)	Mdn (IQR)
	1	2	3	4	5	6	7	8		
Section A: Engagement (Mean)	4.2	4.2	3.0	4.4	3.8	2.8	2.2	2.4	3.4 (0.9)	3.4 (1.5)
Section B: Functionality (Mean)	4.5	3.8	5	4	4.5	3.5	4.8	5	4.4 (0.6)	4.5 (0.9)
Section C: Aesthetics (Mean)	4.3	3.7	3.7	4	5	3.3	2.7	4	3.8 (0.7)	3.9 (0.5)
Section D: Information quality (Mean)	4.7	3.8	3.8	4	5	3.2	3	5	4.1 (0.8)	3.9 (1.1)
App quality (Mean A-D)	4.4	3.8	3.9	4.1	4.6	3.2	3.2	4.1	3.9 (0.5)	4.0 (0.5)
Section E: Subjective Quality (Mean)	4.8	4	4.5	4	4.5	3.8	4	2.8	4.0 (0.6)	4.0 (0.6)
Section F: Perceived Impact (Mean)	5	3.5	3.7	3.2	5	3.3	2.8	3.8	3.8 (0.8)	3.6 (0.8)

Mdn: Median; M: Mean; SD: Standard deviation; IQR: Interquartile range

Dimension	Therapist No.										M (SD)	Mdn (IQR)
	1	2	3	4	5	6	7	8	9	10		
Section A: Engagement (Mean)	4.4	4.6	3.8	4.8	3.8	3.4	3.8	3.4	4.2	3.6	4.0 (0.5)	3.8 (0.7)
Section B: Functionality (Mean)	5.0	4.8	4.0	4.5	4.3	4.0	4.5	4.0	4.0	4.0	4.3 (0.4)	4.1 (0.5)
Section C: Aesthetics (Mean)	4.3	5.0	3.0	5.0	4.0	3.3	4.3	3.3	4.3	4.0	4.1 (0.7)	4.2 (0.8)
Section D: Information quality (Mean)	4.7	3.8	4.0	4.7	3.8	3.8	4.7	4.0	3.6	4.2	4.1 (0.4)	4.0 (0.8)
App quality (Mean A-D)	4.6	4.5	3.7	4.7	4.0	3.6	4.3	3.7	4.0	4.0	4.1 (0.4)	4.0 (0.7)
Section T: Therapeutic benefits (Mean)	4.3	4.7	3.0	5.0	3.8	3.7	4.0	3.7	4.3	4.7	4.1 (0.6)	4.1 (0.9)
Section E: Subjective Quality (Mean)	4	5	3.3	4	3.8	4	3.3	4	3.5	3	3.8 (0.6)	3.9 (0.6)
Section F: Perceived Impact (Mean)	4.5	4.3	3.2	4.7	3.7	4.2	3.3	3.0	3.0	4.2	3.8 (0.6)	3.9 (1.1)

Mdn: Median; M: Mean; SD: Standard deviation; IQR: Interquartile range
